# Environmental Health Intelligence New Zealand (EHINZ): intelligence for public health action

**DOI:** 10.1007/s43999-022-00009-z

**Published:** 2022-09-21

**Authors:** Barry Borman, Carolin Haenfling, Agnieszka Kowalik-Tait, Patrick Hipgrave

**Affiliations:** Environmental Health Intelligence NZ (EHINZ), Research Centre for Hauora and Health, Massey University-Wellington, Wellington, New Zealand

**Keywords:** Spatial analysis, Health indicators, Epidemiology, Health intelligence

## Abstract

The New Zealand health system is data-rich, information-poor, and intelligence meagre. However, there is widespread confusion about the definitions of these terms, so they are often used synonymously. Like many jurisdictions, we continue to collect and collate vast quantities of data at an increasing rate. Many tools are available to “analyse” the data deluge with the false expectation that “intelligence” will be produced. Naively, such a data-driven, machine-analysed paradigm is often thought to produce the “evidence” for decision-making and policy development. Continuing such a blinded approach poses potential health risks to New Zealanders and remains a major impediment to improving our health status

Creating intelligence from information involves humans (perhaps in concert with AI) utilising their interpretative abilities, asking the “so what, “what does it mean” questions, and employing their communication skills to disseminate the answers. To move from information to intelligence requires agencies to employ, develop and maintain a sufficiently skilled workforce over a long period, rather than the quick and easy investment in more and faster machines and software.

Only through a human-driven evaluation of intelligence-based decisions and policies will our knowledge about the environmental health system increase and ultimately yield better health outcomes.

Environmental Health Intelligence NZ (EHINZ) provides intelligence as evidence for decision-making and policy development in environmental health. It is based on the interpretation, communication, and dissemination of information from the surveillance more than seventy environmental health indicators (EHIs) across twelve domains (e.g., air and water quality, climate change), exposure to hazardous substances, and social vulnerability indicators to environmental hazards (e.g., flooding, climate change, sea-level rise, wildfires, heat waves).

The paper details our approach, with two case studies, in providing the NZ health system with “intelligence for environmental health decisions.”

Like many jurisdictions, the New Zealand health system is rich in data, poor in information, and meagre in intelligence. Data of variable quality continues to be collected and collated at an increasing rate. As evident in the COVID-19 pandemic, many tools are available to people with various skills, experience and knowledge to “analyse” these data at great speed and with increasing levels of sophistication. A proliferation of models, dashboards, and other visual aids (eg, graphs, tables etc.) have been developed for analysis and data visualisation. Mistakenly assumption is that such a data-driven, machine-analysed paradigm will produce the “intelligence” or evidence used for decision-making and policy development. Only information, not intelligence, will be produced from data analysis irrespective of its creativity and complexity.

Continuing our blinded, outdated and naïve approach to “intelligence” poses potential health risks to New Zealanders and remains a major impediment to improving our health status.

High-quality, valid data are and will continue to be the critical bedrock of health system information and intelligence. However, many of these data are not only of variable quality and more critically untouched by machines and human thought. Data of dubious quality should be disregarded or cease to be collected. In keeping with the concept of “garbage in, garbage out”, high-quality, sophisticated analysis of low validity data makes a meaningless contribution to a health system. In times of limited resources and increasing demand for health services, decision-makers and policy developers need to use intelligence-based evidence. Creating intelligence from information involves humans (perhaps in concert with AI) utilising their interpretative abilities, asking the “so what, “what does it mean” questions, and employing their communication skills to disseminate the answers. The latter is often a step too far for many agencies, as it requires employing, developing and maintaining a sufficiently skilled workforce over a long period, rather than the quick and easy investment in more and faster machines and software.

Only through the human-driven evaluation of intelligence-based decisions and policies will our knowledge about the health system increase and ultimately yield better health outcomes.

Environmental Health Intelligence New Zealand (EHINZ), established at Massey University in 2009, is primarily funded by the Ministry of Health to provide “intelligence on environment health for public health action”. Its purpose is not data collection but to analyse health-related data, interpret the results of academic research and communicate and disseminate the intelligence through various channels (e.g., websites, factsheets, social media and newsletters).

The EHINZ programme provides added-value intelligence through:

- the surveillance and interpretation of trends in health outcomes linked to environmental hazards and exposures to identify changes over time and identify any emerging issues
- comparing and interpreting the environmental health status of geographic areas and population groups (e.g., age, gender, ethnic group and socioeconomic deprivation) with a focus on vulnerable populations to identify where targeted action is most needed
- the surveillance and interpretation of the effectiveness of policies and other interventions on environmental health, and highlighting good local practice
- raising awareness about environmental health issues, as well as gaps and limitations in environmental health surveillance
- helping to initiate further investigations into the links between the environment and health.

EHINZ has under surveillance more than seventy environmental health indicators (EHIs) across twelve domains (e.g., air and water quality, climate change), exposure to hazardous substances, and social vulnerability indicators to environmental hazards (e.g., flooding, climate change, sea-level rise, wildfires, heat waves).

Various data visualisation tools disseminate our health intelligence to a wide range of stakeholders and clients. Interactive domain-specific dashboards and indicator-specific factsheets are available throughout the website (www.ehinz.ac.nz). For example, the dashboard for the climate change domain displays temperature, and rainfall data, together with several health effect indicators (Fig. 1) at the territorial authority (TA) level. As part of the climate change domain, the energy consumption indicator data are presented at the national level in an interactive indicator-specific factsheet (Fig. 2) using ArcGIS and InstantAtlas software packages [1, 2]. Flourish, a web-based data visualisation tool, has been used to show New Zealand’s mean annual temperature anomaly, 1909-2018 (Fig. 3) [3].

**Fig. 1:**
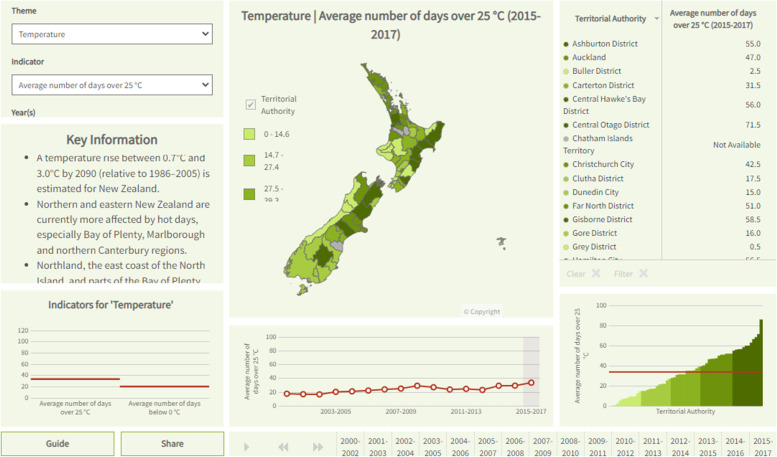
Domain-specific dashboard, Climate Change

**Fig. 2:**
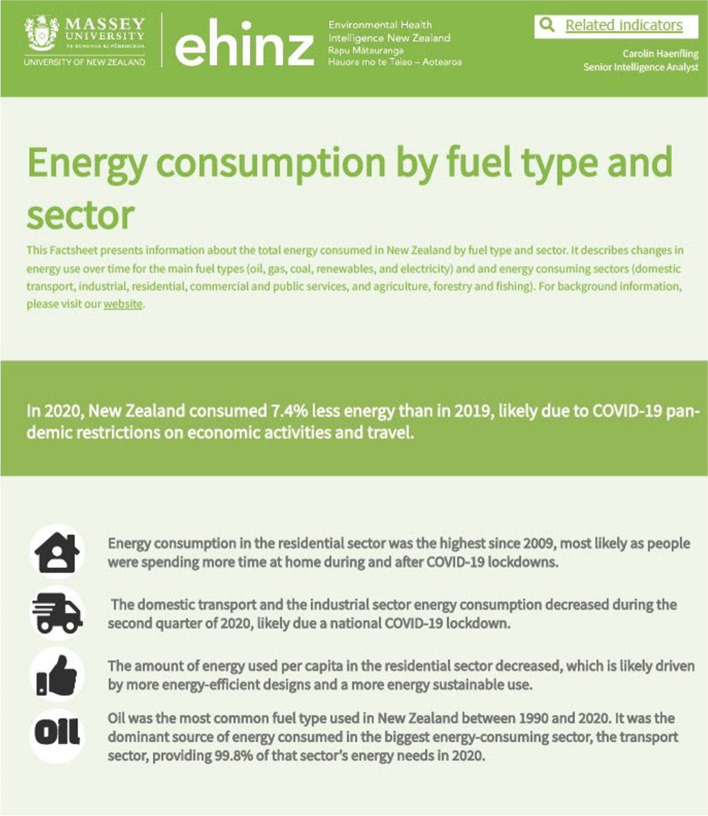
Mean annual temperature anomaly in New Zealand, 1909-2018 (anomaly from the 1981-2010 average temperature across New Zealand)

**Fig. 3:**
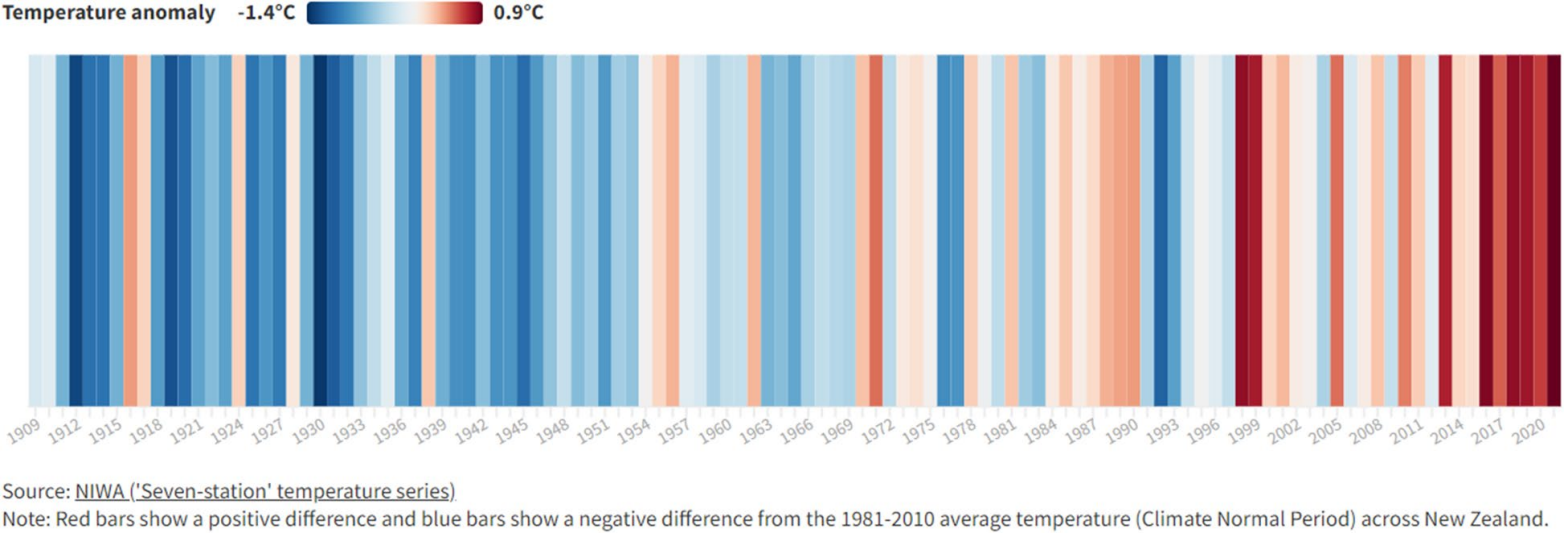
Indicator-specific factsheet/report, Energy consumption (an indicator in the climate change domain)

Healthspace (www.healthspace.ac.nz) allows users to explore the EHIs and other topics through interactive dashboards and local area reports. Again, we use ESRI’s ArcGIS and InstantAtlas to manage large datasets and publish detailed area profiles and easy to understand dashboards. The dashboards and local area reports can display data from various sources at different geographic levels. Topics include: environmental health data (e.g., air and water quality, border health, climate change, population vulnerability, alcohol-related harm), hospitalisations, cancer registrations, mortality, notifiable disease notifications, the New Zealand Health Survey, the national Census of Population and Dwellings, and motor vehicle registrations.

By employing a variety of visualisation tools and interpretating the results, EHINZ can effectively communicate and disseminate environmental health intelligence to stakeholders and clients to benefit the health of New Zealanders.

## Case study 1 – social vulnerability indicators

**Fig. 4:**
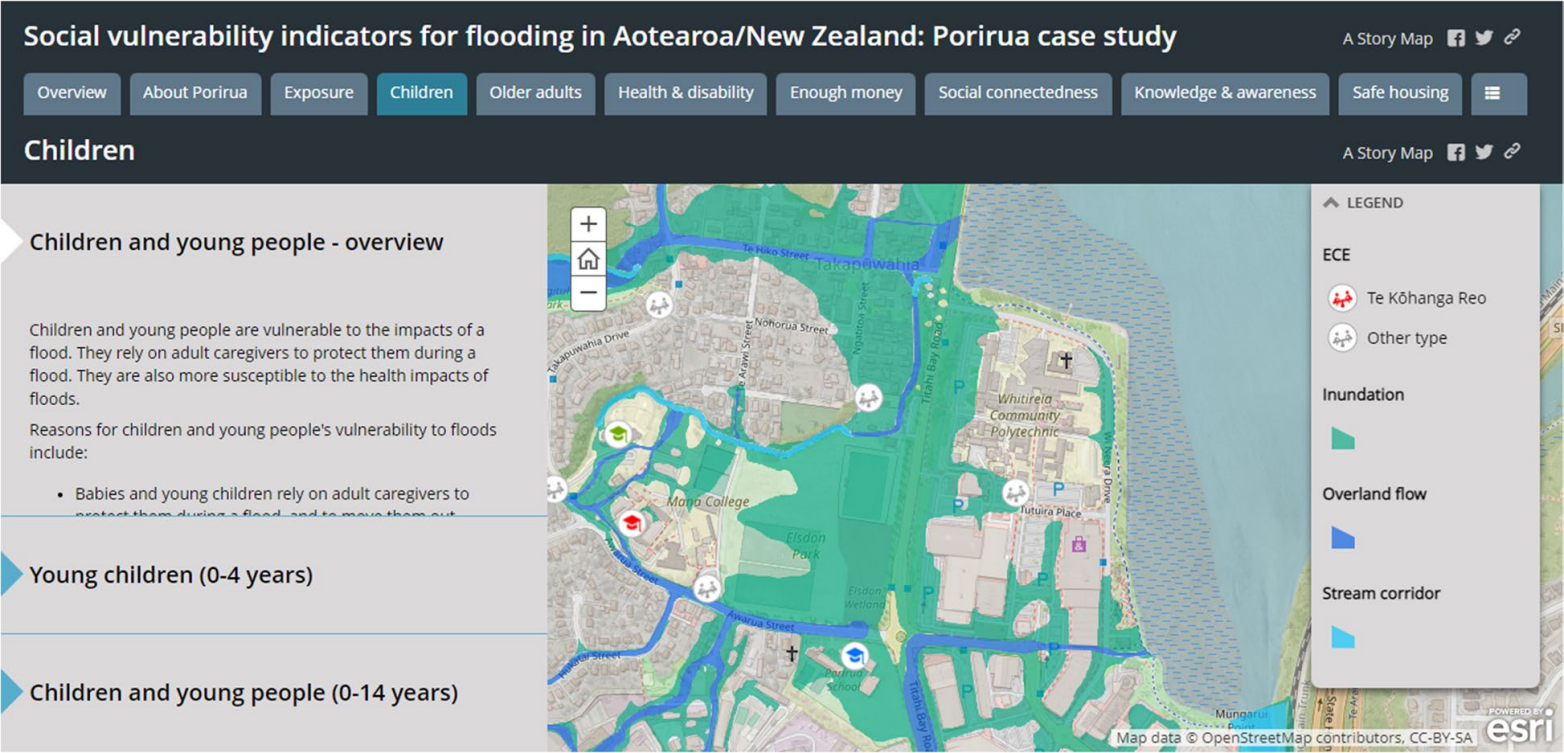
Screenshot of the StoryMap for social vulnerability indicators for flooding, point locations in the children dimension and flood hazard zones

**Fig. 5:**
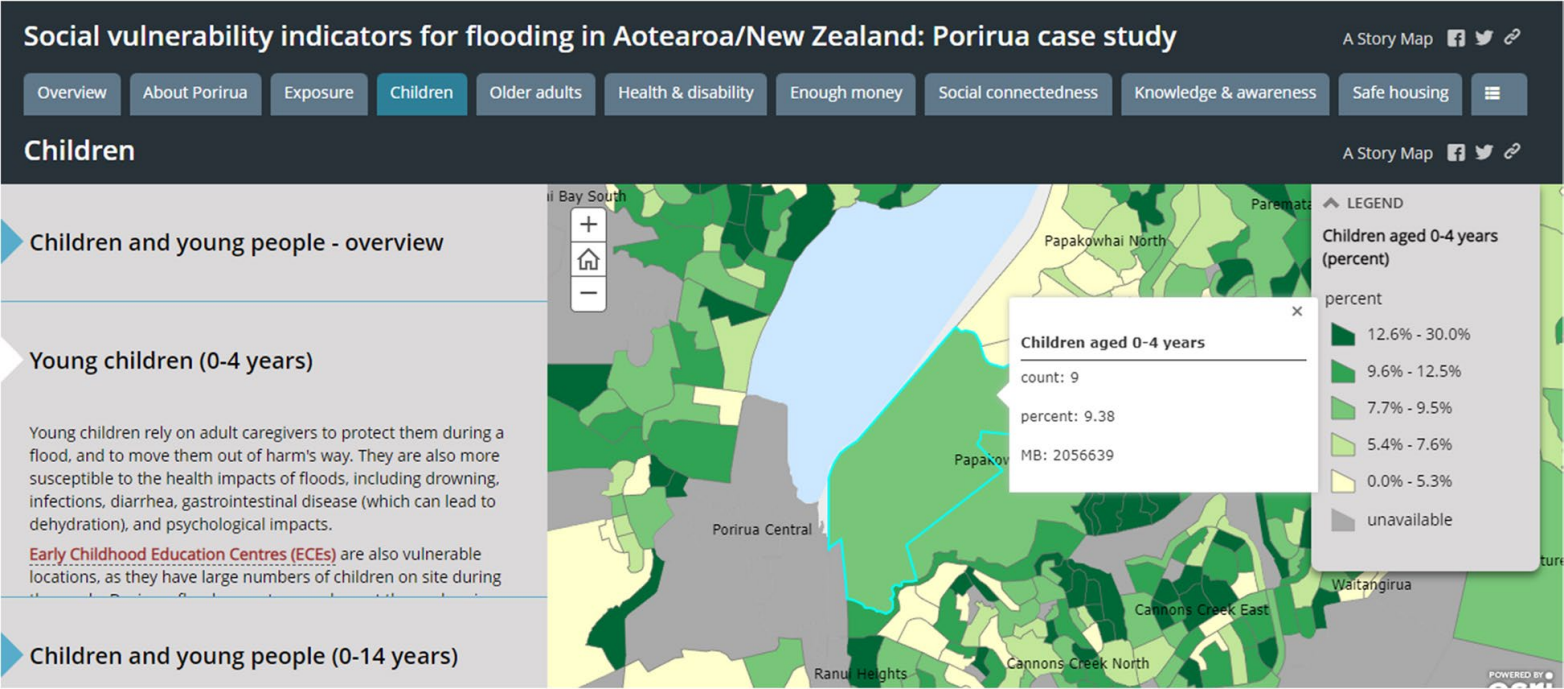
Screenshot of the StoryMap for social vulnerability indicators for flooding, population data in the children dimension

EHINZ developed a set of social vulnerability indicators (SVIs) to identify where people in Porirua City Council area were most vulnerable to the negative impacts of floods (URL: http://www.ehinz.ac.nz/projects/social-vulnerability-indicators/). We were able to identify those areas where people may be less able to anticipate, prepare for, cope with, and recover from a flood. The SVIs included various spatial data such as population data, flood hazard zones, and point locations relating to vulnerable populations (ie, schools, aged care facilities, and health care facilities). ESRI’s ArcGIS StoryMaps was used to visualise the data, which efficiently combines interactive maps (or other content, such as images, videos, or graphs) and short narratives to illustrate spatial relationships and tell a compelling story [4] (Fig. 4). Indicators were presented for each dimension of social vulnerability (eg, children, social connectedness, safe housing, etc), with a rationale about why they are essential for social vulnerability. Figure 4 is a StoryMap screenshot displaying the locations of schools, early childhood centres (ECE) and potential flood hazard zones, which aids in identifying schools and ECE that are more at risk from flooding. The interactive map provides vital information for possible evacuation efforts on the type of school and Early Childhood Centres, and the number of children enrolled. Other maps in the children dimension display the number and percentage of children (0–4-year-olds, and 0–14-year-olds) by meshblock (MB, the smallest geographic unit for which data is collected and processed by Stats NZ) in the Porirua City area (Fig. 5).

**Fig. 6:**
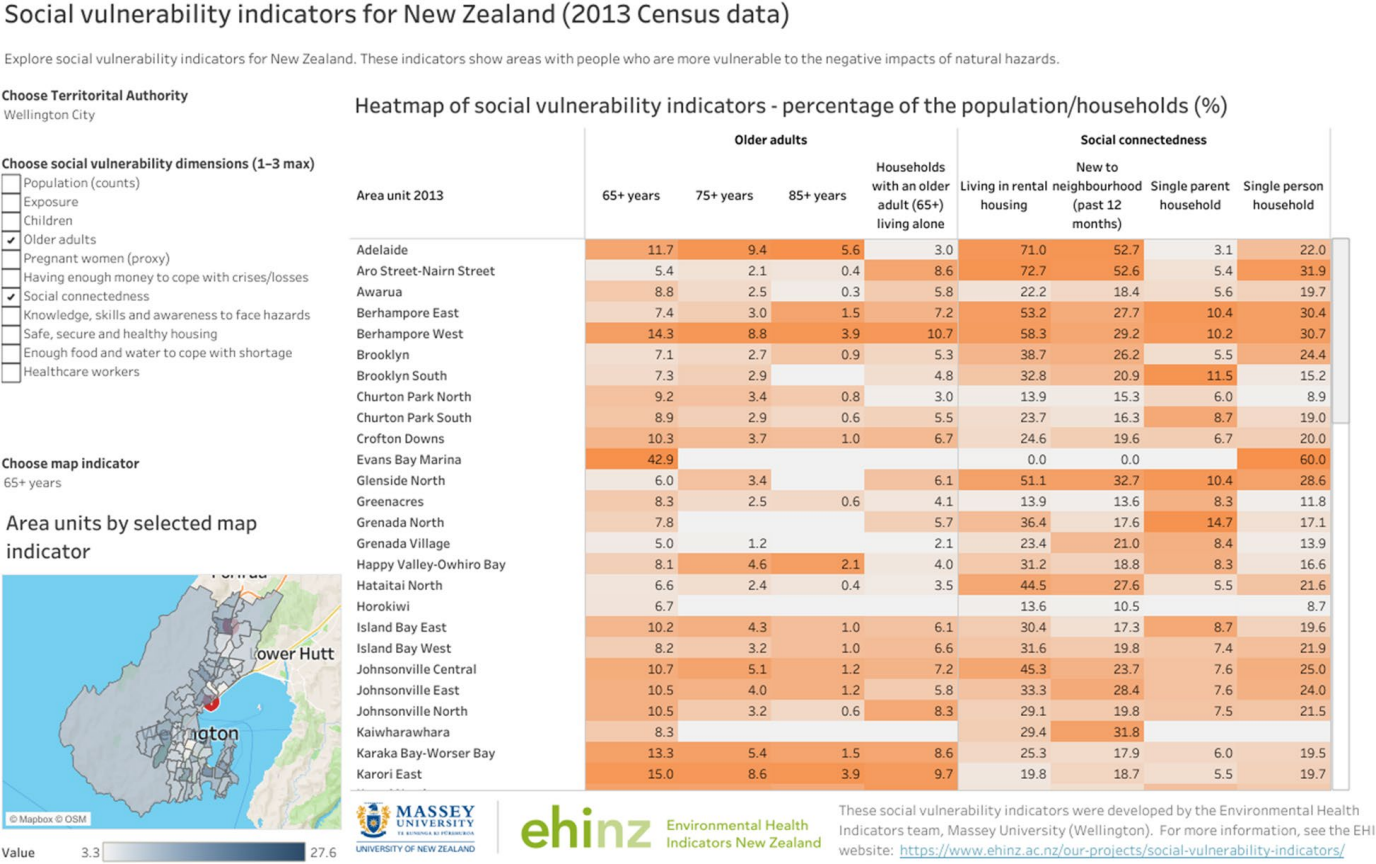
Screenshot of the Data Explorer tool for social vulnerability indicators for flooding, children dimension

The Data Explorer tool using Tableau [5] (Fig. 6) displayed the SVIs at the national level in interactive tables and heatmaps at various geographic levels. The heatmaps can effectively show areas with higher or lower vulnerability to flooding and summarise the indicators across all dimensions of social vulnerability.

In 2020, the project was expanded to incorporate social vulnerability indicators to other natural hazards as well as pandemics (URL: http://www.ehinz.ac.nz/indicators/population-vulnerability/social-vulnerability-to-natural-hazards/).

## Case study 2 – identifying alcohol hotspots

At the request of several local councils, we were able to produce maps of the density of licensed premises where alcohol is served. With these maps, client agencies can quickly identify hotspots of high licence density or concentrations of a particular type of licence and use this information to inform alcohol-related policy or public health interventions by highlighting where the effects of alcohol are likely to be most keenly felt.

**Fig. 7:**
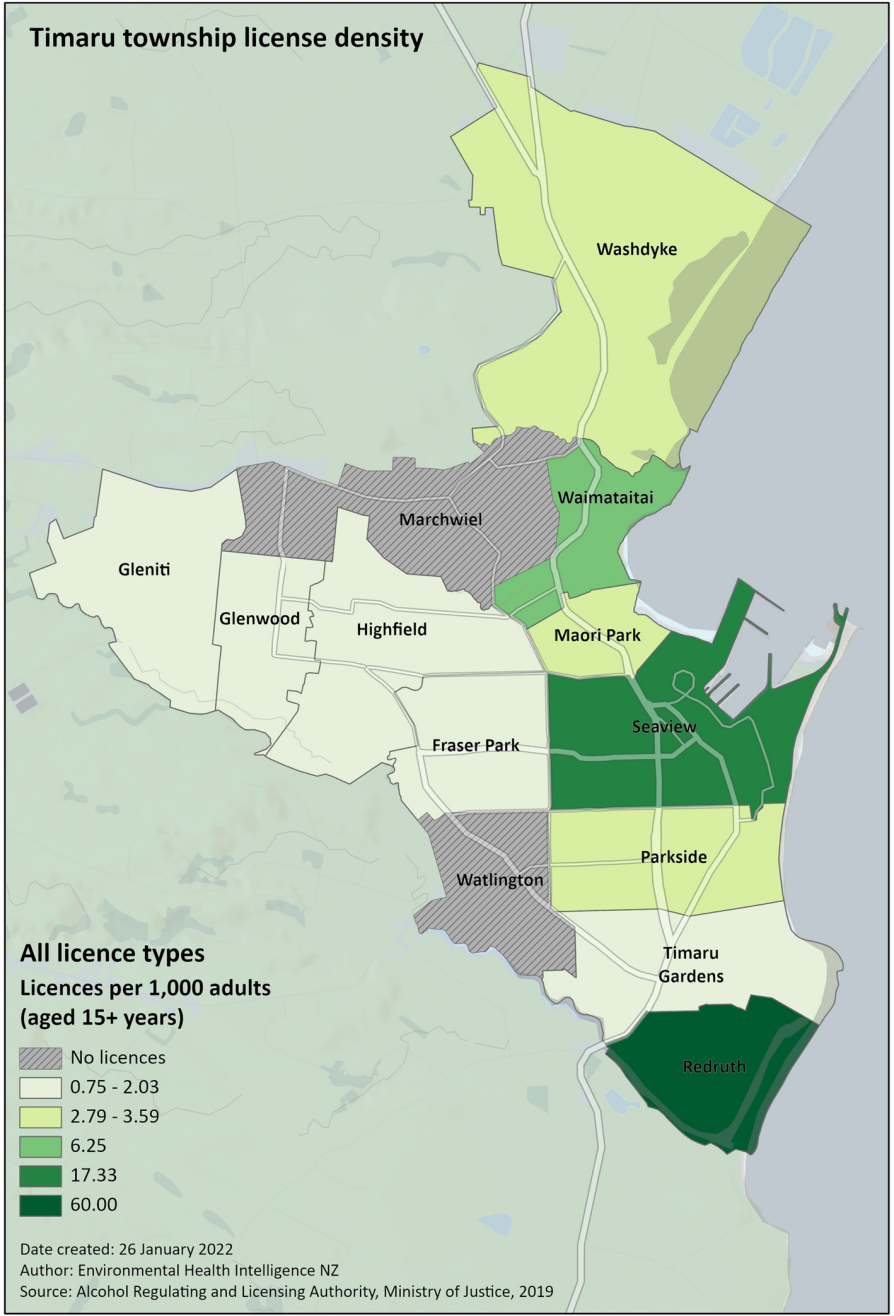
Thematic map of alcohol license density, by area unit, in Timaru

**Fig. 8:**
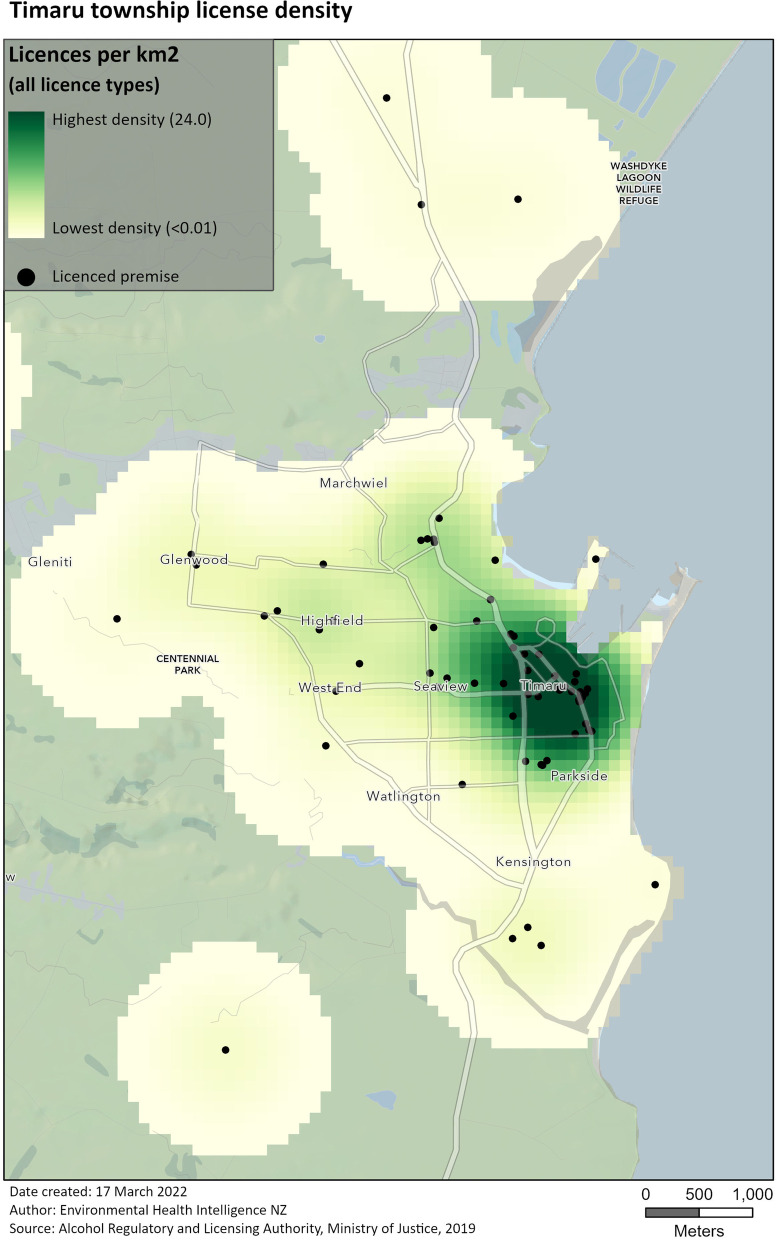
Kernel density map of licence density in Timaru

We use two styles of mapping using ArcGIS Pro [1]  to present the information:

- Thematic maps¸ which show the number of licences per 1,000 adult residents of area units (AUs) within the area of interest (Fig. 7). While these maps effectively communicate the relationship between the number of people and licences in specific areas, they are less effective at communicating the concentration of alcohol outlets across space.
- For this reason, Kernel density maps are also used, the better to show the concentration of the licenced premises themselves (Fig. 8). This helps distinguish areas that have a high per-population density because they contain a large number of licences from those that have one because of a small population - compare the Redruth area unit in Fig. 7 to the same area in Fig. 8.

Perhaps more importantly, the kernel density maps do not constrain themselves to defined geographical areas as many other maps do. This is a useful trait in this case, because residents of a given AU are unlikely to be affected only by the presence of alcohol outlets within the borders of their own AU.
